# Pregabalin and Radicular Pain Study (PARPS) for Cervical Spondylosis in a Multiracial Asian Population

**DOI:** 10.4021/jocmr879w

**Published:** 2013-12-13

**Authors:** Yew Long Lo, Priscilia Woon Ting Cheong, Jane Mary George, Seang Beng Tan, Wai Mun Yue, Chang Ming Guo, Stephanie Fook-Chong

**Affiliations:** a Department of Neurology, National Neuroscience Institute, Singapore General Hospital, Outram Road, Singapore 169608, Singapore; b Department of Neurology, Singapore General Hospital, Outram Road, Singapore 169608, Singapore; c Department of Anesthesiology, Singapore General Hospital, Outram Road, Singapore 169608, Singapore; d Department of Orthopedic Surgery, Singapore General Hospital, Outram Road, Singapore 169608, Singapore; e Department of Clinical Research, Singapore General Hospital, Outram Road, Singapore 169608, Singapore

**Keywords:** Cervical spondylosis, Cervical radiculopathy, Cervical myelopathy, Pregabalin, Trial, Asian

## Abstract

**Background:**

Pain from cervical spondylosis (CS) may result from degenerative spinal canal stenosis (cervical spondylotic myelopathy (CSM)) or lateral recesses compromise, leading to nerve root compression (cervical spondylotic radiculopathy (CSR)). Pregabalin was shown to be effective in randomized, placebo-controlled trials for post-herpetic neuralgia and diabetic neuropathy. We evaluate its efficacy in CS with underlying CSR or CSM in a prospective study comprising Asian patients for the first time.

**Methods:**

Patients with CS and CSR or CSM (clinical, MRI, or electrophysiological evidence) presenting with neuropathic pain were recruited. We excluded patients with diabetes, underlying neurological disease or who were previously on antiepileptics. Pregabalin 75 mg bd was administered for 4 weeks, after which dosage was increased to 150 mg bd for another 4 weeks if the visual analog scale (VAS) was not reduced by 50%. In addition, we monitored the short form McGill pain questionnaire (SFMPQ) at baseline, 4 weeks and 8 weeks. Mood changes were monitored using the hospital anxiety and depression score (HADS) with an identical timeline.

**Results:**

We recruited 50 patients, of which 23 completed the trial. Of the 27 who withdrew, 12 (44%) were for somnolence. Thirteen patients’ (54%) dosages remained at 75 mg and 11 patients’ (46%) dosages were escalated to 150 mg bd. There were significantly reducing trends from baseline for VAS (ANOVA, F_(1, 21)_ = 25.4, P < 0.0005), SFMPQ (sensory) (F_(1, 22)_ = 11.2, P = 0.003), and SFMPQ (affective) (F_(1, 21)_ = 10.9, P = 0.008). For VAS, there was significant reduction at 4 weeks (P = 0.001) and 8 weeks (P < 0.0005) compared to baseline. For SFMPQ (sensory), there was significant reduction at 4 weeks (P = 0.01) and 8 weeks (P = 0.006) in scores compared to baselines. For SFMPQ (affective), there was significant reduction at 4 weeks (P = 0.04) and 8 weeks (P = 0.008) in scores compared to baseline. No significant anxiety (F_(1, 4)_ = 1.3, P = 0.32) or depression (F_(1, 4)_ = 0.06, P = 0.82) changes were observed in the HADS.

**Conclusion:**

Pregabalin is efficacious in alleviation of pain symptoms related to CSR as a first-line single agent, evaluated by quantitative severity and other experiential scales. No significant mood changes reported in other studies were demonstrated. Somnolence was commonest adverse effect leading to high dropout rates, occurring early even at the lowest dose. The findings suggest the need for further studies of efficacy at lower dosages, particularly in the Asian population.

## Introduction

Cervical spondylosis (CS) is characterized by degenerative lateral recesses compromise, leading to nerve root compression (cervical spondylotic radiculopathy (CSR)), or more severe spinal canal stenosis and cord compromise (cervical spondylotic myelopathy (CSM)). Medical management is usually the initial step, often consisting of pharmacological treatment and rehabilitation [1, 2]. The medical management of neuriopathic pain in CS can be clinically challenging.

Pregabalin is structurally related to the anti-epileptic drug gabapentin. It binds strongly and selectively to the alpha-2-delta subunit of hyper-excited voltage-gated calcium channels, leading to a reduction in calcium influx and synaptic release of excitatory neurotransmitters. This mechanism is believed to be responsible for its analgesic and anticonvulsant properties [3, 4].

Pregabalin has demonstrated efficacy in randomized, placebo-controlled trials for post-herpetic neuralgia and diabetic neuropathy [5, 6]. Its widespread, potent action and lack of drug interactions render it suitable for pain management. However, its efficacy in CS, especially for patients with CSR-associated pain, has not been established. Additionally, there are no Asian studies of pregabalin usage in neuropathic pain published to date.

In this study, we evaluate its efficacy for treating neuropathic pain in degenerative CSR/CSM patients in a prospective fashion for the first time. Self-evaluation and investigator-rated scorings were implemented to ascertain effectiveness and tolerability of pregabalin in a multi-racial Asian setting.

## Methods

The patients were recruited prospectively from a general neurology service in a tertiary hospital. They were referred for diagnosis or management of complaints relating to the cervical spine. We included patients presenting with neck pain, which may be associated with numbness or weakness in a cervical root distribution. Patients should have signs of dermatomal sensory loss or motor weakness, reflex changes or even myelopathy. All patients must have imaging evidence (plain radiograph or MRI) of CS. We excluded patients with diabetes mellitus, chronic renal impairment or neurological disorders presenting with polyneuropathy which may otherwise confound assessment of outcomes. The local ethics committee has approved the study protocol, which has also been registered with clinicaltrial.gov (identifier number: NCT01061697).

Pregabalin was commenced at 75 mg twice a day for a 4-week period, after which patients returned for follow-up. If the VAS pain scale was not reduced by 50%, the dosage was increased to 150 mg twice a day for a further 4 weeks. Adverse events to the study drug were documented carefully. Apart from physiotherapy, patients who had prior usage of pain prophylactic agents, including antiepileptics, gabapentin, and pregabalin were excluded. Of the original 50 recruited patients, 12 were on paracetamol and 10 were on indomethacin on an *ad-hoc* basis. As these were short-acting agents, they were discontinued once the trial commenced. During the trial period, none of the patients had any other form of analgesia.

Each patient observed symptoms for 1 week prior to starting medication to obtain average baseline score values. We assessed pain symptoms on an 11-point visual analog scale (VAS) as the primary outcome measure. In addition, the short form McGill pain questionnaire (SFMPQ), comprising both sensory and affective components, was the main secondary outcome measure [7]. SFMPQ (sensory) consisted of 11 components and SFMPQ (affective) consisted of 4 components. Patients scored each component either as none (0), mild (1), moderate (2) or severe (3). Patients were instructed to obtain a daily diary and make records on awakening based on the average perceived pain over the last 24 h. Average values of VAS and SFMPQ scores were obtained upon review at 4 and 8 weeks after commencement. At the end of the 8-week study, the clinical global impression of change (CGIC) and patient global impression of change (PGIC) scales were administered. In view of FDA alerts in relation to mood changes in antiepileptic agents, baseline hospital anxiety and depression scores (HADS) were recorded at baseline, 4 weeks, and 8 weeks after commencing pregabalin [8].

Statistical analysis was completed with SPSS for Windows package. A P value obtained at < 0.05 denoted statistical significance.

## Results

We recruited 50 patients (mean age: 51.2 years; range: 24 to 69 years; 25 men; 40 Chinese, 5 Indians, 2 Malays, and 3 Eurasians), of which 23 completed the trial. Of the 27 who withdrew, 12 (44%) were for somnolence. Thirteen patients’ (54%) dosages remained at 75 mg and 11 patients’ (46%) dosages were escalated to 150 mg bd. Of the 28 patients who completed 4 weeks of trial, 13 (46%) had experienced > 50% reduction in pain. Based on an intention-to-treat analysis pertaining to the proportion of the original 50 patients recruited, 13 patients (26%) experienced > 50% reduction in pain.

There was no significant difference in age and weight when comparing patients who completed or dropped out of the study (unpaired t-test, P > 0.05). The liver function tests were not routinely monitored during the trial as pregabalin [9] is not subject to hepatic metabolism nor affect liver enzyme systems such as cytochrome P450. None of our patients had history of renal insufficiency necessitating dose adjustment.

**Figure 1:**
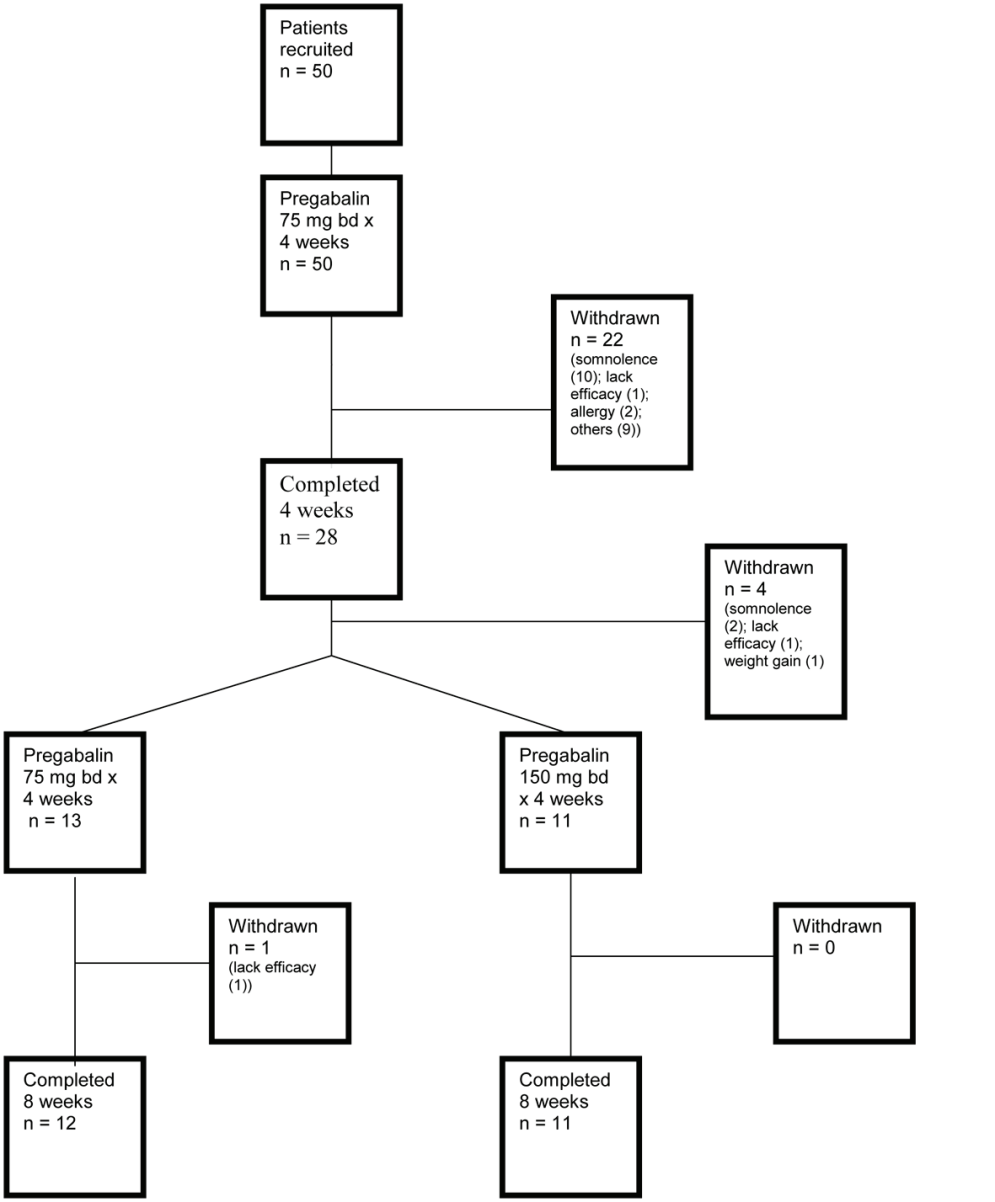
Flow diagram depicting patient recruitment from iniation to end of the trial process.

Figure 1 is a flow diagram depicting the entire trial process.

There were significantly reducing trends from baseline for VAS (ANOVA, F_(1, 21)_ = 25.4, P < 0.0005), SFMPQ (sensory) (F_(1, 22)_ = 11.2), P = 0.003), and SFMPQ (affective) (F_(1, 21)_ = 10.9, P = 0.008). For VAS, there was significant reduction at 4 weeks (P = 0.001) and 8 weeks (P < 0.0005) compared to baseline. Additionally, repeated contrast testing of VAS at each week compared to the previous revealed significant reduction up to week 2. No further reduction was evident beyond week 2. Paired t-test was used to compare VAS scores between week 4 (after which dosages were maintained or doubled) and week 8. This showed a modest reduction in mean VAS of approximately 0.62 (P = 0.04). Comparing responses from week 4 to week 8 (paired t-test), the 75 mg bd (P = 0.24) arm did not show significant additional pain reduction. However, the 150 mg bd (P = 0.04) arm showed mild additional reduction in VAS scores.

For SFMPQ (sensory), there was significant reduction at 4 weeks (P = 0.01) and 8 weeks (P = 0.006) in scores compared to baselines. For SFMPQ (affective), there was significant reduction at 4 weeks (P = 0.04) and 8 weeks (P = 0.008) in scores compared to baseline.

The PGIC scoring of patients who completed the study showed that 13% experienced no change in symptoms, 43% having minimal improvement, 30% having much improved, and 13% experiencing very much improvement. Similarly, the CGIC scoring showed that 22% experienced no change in symptoms, 35% having minimal improvement, 35% having much improved, and 22% experiencing very much improvement.

No significant anxiety (F_(1, 4)_ = 1.3, P = 0.32) or depression (F_(1, 4)_ = 0.06, P = 0.82) changes were observed in the HADS.

For the most common adverse effects of somnolence, 33 of 34 patients already experienced it at 75 mg bd dosage, and only 1 more patient had complained of its onset at the 150 bd dose stage.

**Figure 2:**
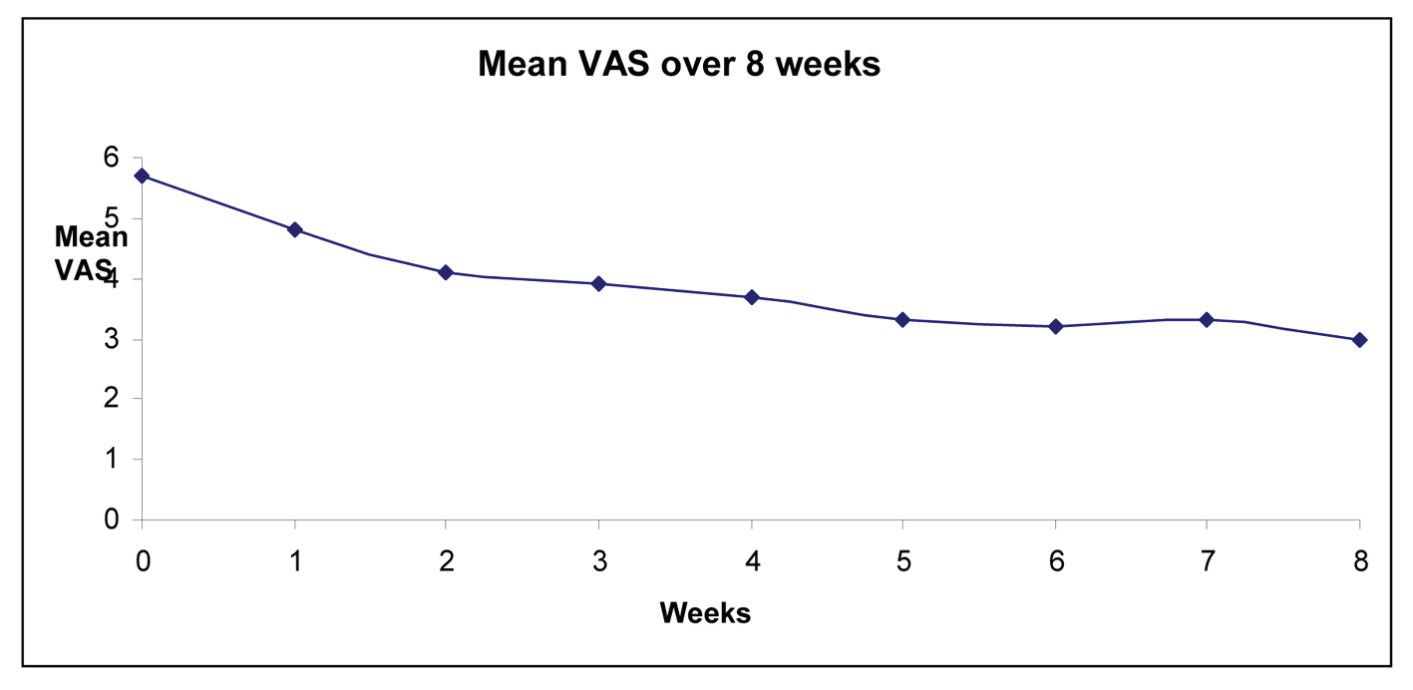
Graphical illustration of mean pain reduction on the VAS scale over time. Mean values were: 5.7 (week 0 or baseline), 4.8 (week 1), 4.1 (week 2), 3.9 (week 3), 3.7 (week 4), 3.3 (week 5), 3.2 (week 6), 3.3 (week 7), and 3 (week 8). Significant benefit was achieved by the 2nd week after pregabalin initiation.

Figure 2 shows time and dose responses in a graphical fashion. Table 1 summarizes patients’ adverse effects.

**Table 1:** Summary of Adverse Effects of 42 Patients

Adverse effects	Number (%)
Somnolence	34 (80.9)
Dizziness	14 (33.3)
Headache	5 (11.9)
Weight gain	6 (14.3)
Dry mouth	7 (16.7)
Blurred vision	5 (11.9)
Irritability	3 (7.1)
Forgetfulness	3 (7.1)
Increased appetite	3 (7.1)
Attention disturbance	2 (4.8)
Confusion	1 (2.4)
Rash	1 (2.4)
Eye swelling	1 (2.4)
Lethargy	1 (2.4)

## Discussion

This is the first Asian study of pregabalin for treating neuropathic pain in cervical spine disorders to our knowledge. It was designed as a prospective and observational trial as pregabalin has already been shown to be useful for treating neuropathic pain in Western settings [5, 6]. Here, we show that it is efficacious for alleviation of pain symptoms related to CSR. The beneficial effects were evident at 2 weeks after pregabalin was commenced. Of note, significant proportion of patients (46%) experienced > 50% of pain reduction after 4 weeks of treatment. These findings point to a fairly rapid onset of action in the initial trial phase, consistent with the experience of other investigators [7]. The beneficial effects were evident at 4 weeks and persisted onto 8 weeks after trial iniation. In addition to physical rating of pain in the VAS scale, there were significant reduction in secondary outcome measures, notably the SFMPQ, which evaluates qualitative aspects of pain in greater detail.

Of the patients that continued medication after the initial 4 weeks (Fig. 1), there was no significant pain reduction at the 8-week time point with the 75 mg bd dose. However, there was a mildly significant benefit for the 150 mg bd arm (P = 0.05). This suggests that in Asians, a higher pregabalin dose did not show a clear benefit in non-responders at the lower dose, but a larger patient number would be justified to validate this finding. Importantly though, higher dosing should be balanced with the possibility of adverse events mentioned below.

In comparison, previous pregabalin trials for neuropathic pain [5, 6] utilizing dosages up to 600 mg/day and permitting narcotic or non-narcotic analgesics, antidepressants, and even recent usage of gabapentin, we had strictly excluded all these conditions. This was to allow for better characterization of efficacy, with view to pregablin’s role as a first-line or single treatment agent. However, the main drawback would be limitation of the number of patients eligible for recruitment. Other studies had investigated its role in post-traumatic neuropathic pain [10], allodynia [11], and central pain relating to spinal cord injury [12]. All had concluded efficacy in the range of 150 to 600 mg a day. These were significantly higher than dosages employed in the present trial.

There were no serious adverse events observed in this trial, such as anaphylaxis, cardiovascular collapse or gastrointestinal complaints. In spite of FDA alerts on mood changes associated with antiepileptic drugs [13], our HADS scores did not suggest any significant anxiety or depression over the 8-week trial period.

Of the documented adverse events, somnolence and dizziness were by far most commonly observed, constituting 81% and 33% respectively. These proportions were much higher than those reported in two large pregabalin trials, which were in the 25% range [5, 6]. While it is known that Asians require lower dosages of analgesic agents compared with Europeans [14] possibly due to pharmacokinetic or pharmacodynamic differences, the underlying mechanism remains unclear. To our knowledge, there are no published studies formally addressing the relation of somnolence as a side effect in Asians in relation to their Western counterparts. It is pertinent to note that a large proportion of withdrawals were observed in earliest stage of the trial when patients were administered 75 mg bd (Fig. 1), suggesting that the initiation dose played an important role in patient tolerance. As anti-epileptic agents may require at least 1 to 2 weeks for optimal efficacy, a lower initial dosage would likely influence patient behavior significantly. At present, available formulations are 75 mg and 150 mg; as such, our findings suggest that provision of lower dosage formulations would be of value, particularly in the Asian context.

The present study is limited by the lack of placebo, small sample size and heterogeneous patient study group comprising Chinese, Malay, and Indians. While the high patient drop-out rates were observed, they were not apparently related to age or weight. This suggests that other pharmacological or intrinsic patient factors may be responsible for this observation. Nonetheless, these findings may provide additional justification of a trial at lower starting dosages in Asians.

In conclusion, this PARPS has shown that pregabalin has rapid onset and is efficacious for neuropathic pain related to CS as a first-line single agent. However, the high proportion of adverse effects justifies further investigations into a lower initial dosage for Asian patients.
